# Metabolome signature of autism in the human prefrontal cortex

**DOI:** 10.1038/s42003-019-0485-4

**Published:** 2019-06-21

**Authors:** Ilia Kurochkin, Ekaterina Khrameeva, Anna Tkachev, Vita Stepanova, Anna Vanyushkina, Elena Stekolshchikova, Qian Li, Dmitry Zubkov, Polina Shichkova, Tobias Halene, Lothar Willmitzer, Patrick Giavalisco, Schahram Akbarian, Philipp Khaitovich

**Affiliations:** 1 Center for Neurobiology and Brain Restoration, Skolkovo Institute of Science and Technology, Moscow, 121205 Russia; 2 Institute for Information Transmission Problems, Russian Academy of Sciences, Bolshoy Karetny Per. 19/1, Moscow, 127051 Russia; 3 CAS Key Laboratory of Computational Biology, CAS-MPG Partner Institute for Computational Biology, 320 Yue Yang Road, 200031 Shanghai, China; 4 Department of Psychiatry and Friedman Brain Institute, Icahn School of Medicine at Mount Sinai, New York, NY 10029 USA; 5 Max Planck Institute for Molecular Plant Physiology, Am Mühlenberg 1, Potsdam, 14476 Germany; 6 Max Planck Institute for Biology of Ageing, Cologne, 50931 Germany; 7 Max Planck Institute for Evolutionary Anthropology, Deutscher Platz 6, Leipzig, 04103 Germany

**Keywords:** Autism spectrum disorders, Molecular neuroscience, Metabolomics

## Abstract

Autism spectrum disorder (ASD) is a common neurodevelopmental disorder with yet incompletely uncovered molecular determinants. Alterations in the abundance of low molecular weight compounds (metabolites) in ASD could add to our understanding of the disease. Indeed, such alterations take place in the urine, plasma and cerebellum of ASD individuals. In this work, we investigated mass-spectrometric signal intensities of 1,366 metabolites in the prefrontal cortex grey matter of 32 ASD and 40 control individuals. 15% of these metabolites showed significantly different intensities in ASD and clustered in 16 metabolic pathways. Of them, ten pathways were altered in urine and blood of ASD individuals (Fisher test, *p* < 0.05), opening an opportunity for the design of new diagnostic instruments. Furthermore, metabolic measurements conducted in 40 chimpanzees and 40 macaques showed an excess of metabolite intensity differences unique to humans, supporting the hypothesized disruption of evolutionary novel cortical mechanisms in ASD.

## Abstract (web summary)

Kurochkin, Khrameeva et al. show differences in metabolite intensities within the prefrontal cortex of individuals with autism spectrum disorders (ASD) that converge into 16 cellular pathways. Parallel measurements conducted in primates also revealed unique metabolite intensity differences unique to humans, highlighting unique evolutionary cortical mechanism in ASD.

## Introduction

Autism spectrum disorder (ASD) is characterized by the developmental disruption of complex cognitive traits, especially those underlying communication and socialization. These traits are particularly developed in humans, suggesting a disruption of recently evolved cognitive mechanisms in ASD^1–3^. This notion is supported by the assessment of gene expression patterns during cortical development in healthy humans and ASD individuals in comparison to nonhuman primates^4^.

ASD is a heterogeneous condition characterized by a broad spectrum of symptom severity^5^. Accordingly, despite the reported high heritability of the disease^6–8^, numerous genetic studies indicate that most ASD cases are likely to be caused by a large number of genetic variants, each of which has a small effect^9,10^.

In the absence of clear genetic determinants, investigations looked for potential biochemical landmarks of disease by monitoring concentrations of low weight molecular compounds (metabolites) in urine, blood plasma and the brain. To date, metabolite concentration differences in the urine of ASD individuals have mostly been studied using systematic metabolomics profiling techniques, which yielded results for 20 to 622 metabolites measured in 14 to 48 ASD individuals and comparable control groups^11–22^. The pioneering study conducted by Yap et al.^11^, using proton-nuclear magnetic resonance (NMR) and involving 39 ASD individuals demonstrated alterations of amino acid metabolism and the tryptophan and nicotinic acid metabolic pathways. Similarly, another study by Ming et al.^12^ identified differences in amino acid metabolism, as well as metabolic signatures of oxidative stress, in the urine of 48 children with ASD using a combination of liquid chromatography and gas chromatography coupled with mass spectrometry (LC–MS and GC–MS). Changes in the purine metabolism pathway, pyrimidine metabolism pathway and changes in several pathways involved in the metabolism of tyrosine, asparagine, tryptophan and arginine were further identified in the urine of 30 ASD individuals using a combination of the NMR and LC–MS approaches were reported by Dim et al.^19^. Most recently, Bitar et al.^22^, employing a combination of the NMR and LC–MS approaches, reported concentration changes of metabolites related to oxidative stress, including glutathione metabolism, changes in cysteine, methionine, arginine, and proline metabolism pathways, as well as changes in carbohydrate metabolism, including the metabolism of propanoate, citrate, and sugars in the urine of 40 ASD individuals.

The first study investigating metabolite concentration differences in blood identified changes associated with mitochondrial dysfunction, as well as various metabolic pathway changes, such as a disruption in the tricarboxylic acid (TCA) cycle in the plasma samples of 52 children diagnosed with ASD using five mass spectrometry-based methods were reported by West et al.^23^. In a more recent study, Wang et al.^24^ identified the concentration differences of 17 metabolites in the blood plasma of 73 ASD individuals. Of them, 11 metabolites, including sphingosine 1-phosphate and docosahexaenoic acid, were validated in an independent cohort^24^.

The only study conducted in the brain, by Graham et al.^25^, identified concentration differences of 37 metabolites in the cerebellum of 11 ASD individuals and 11 controls using LC–MS. These differences were not enriched in any biological pathway^25^.

In addition to studies assessing the concentration levels of multiple metabolites, others focused on particular compounds, such as markers of mitochondrial dysfunction that are believed to be associated with the disease^26–28^. These studies reported an elevated concentration of glutamate^29,30^, as well as glycolysis products, such as lactate and pyruvate, in the serum of ASD individuals^31^. Other findings included a decreased concentration of carnitine, the fatty acid carrier from the cytosol to the mitochondria^32^, and glutathione, the reported key reactive oxygen species neutralizer, in ASD individuals’ blood^33^. By contrast, the concentration of palmitate, one of the key energy sources for mitochondria, was increased in ASD plasma samples^34^.

Even though the existing studies provided substantial coverage of the metabolite concentration differences detected in ASD individuals in urine and blood, little is known about the relationship between these differences and metabolic alterations taking place in the brain. The only study performed in the brain focused on cerebellum and involved a limited number of individuals. In our study, we were able to compare metabolite intensity differences detected in the prefrontal cortex (PFC) of the brain with differences detected in urine and blood by the preceding studies. Specifically, we investigated changes in intensities of 1366 metabolites detected using untargeted LC–MS in the prefrontal cortex gray matter of 40 control and 32 ASD individuals. We further analyzed the metabolite intensities in the PFC of 40 chimpanzees and 40 macaques to investigate the relationship between metabolic alterations in ASD and brain metabolic features unique to humans. This might be informative, as ASD affects an array of cognitive functions particular to the human brain. We identified numerous metabolite intensity changes distinguishing ASD individuals from the controls. Notably, the majority of these differences clustered in metabolic pathways previously identified in the urine and blood of ASD individuals. Furthermore, several of these pathways contained an excess of metabolite intensity changes unique to humans, indicating a disruption of evolutionary novel cortical metabolic features in ASD.

## Results

### Metabolic data description

We searched for metabolic alterations in brains of ASD individuals by measuring intensities of polar low-molecular-weight compounds in the postmortem PFC samples of 40 controls and 32 ASD individuals (Supplementary Data 1). The control and ASD samples were matched for sex and sample quality, which were estimated using RNA integrity levels. Each group covered a broad age range: 2–60 years for ASD individuals and 0–61 years for controls (Supplementary Data 1). All samples were represented by the cortical gray matter.

**Fig. 1:**
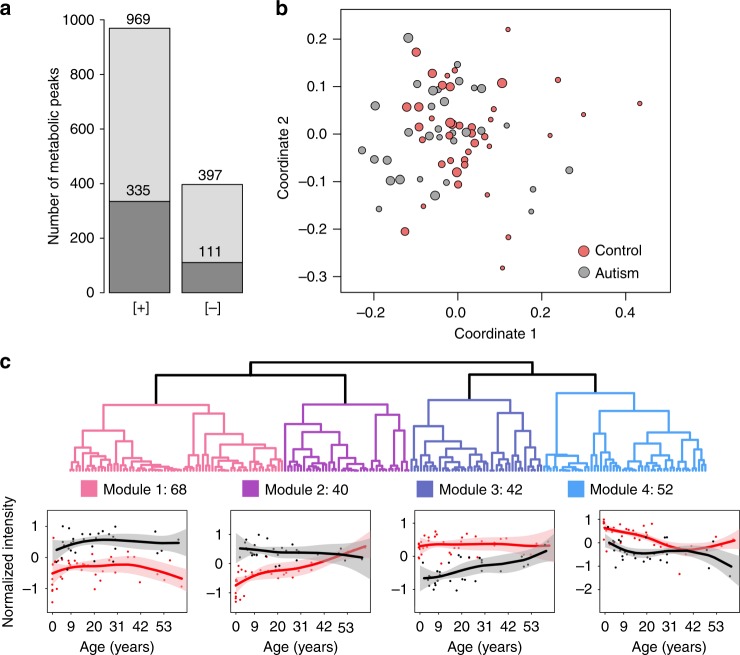
Identification of metabolite intensity differences in the prefrontal cortex of ASD individuals. **a** The number of metabolite peaks detected by LC–MS procedure in positive ([+]) and negative ([−]) ionization modes, after the removal of peaks affected by confounding factors. Darker shades indicate metabolite peaks putatively annotated using probabilistic matching to the LIPID MAPS database and HMDB. **b** The relationship among individuals plotted as the first two dimensions of the multidimensional scaling (MDS) procedure based on intensities of 1366 metabolites. Circles represent individual samples. Colors represent ASD individuals (gray) and control individuals (red). The size of each circle is proportional to the individual’s age (smaller circles correspond to younger ages). **c** Hierarchical clustering dendrogram based on intensities of 202 ASD-related metabolites and intensity patterns in the four cluster modules. The metabolite intensities within each module were standardized to mean = 0 and standard deviation = 1. Points represent mean intensities in each individual (red—controls; black—ASD). Lines show cubic spline curves fitted to the data. Pink and gray shaded areas show one standard deviation of the curve estimates

The measurements, conducted in a random order using liquid chromatography coupled with mass spectrometry (LC–MS) in positive and negative ionization modes, yielded 4065 and 1685 distinct MS peaks representing polar compounds (metabolites) with molecular weights below 2000 Da. Among them, 801 and 209 metabolites were putatively annotated using probabilistic matching to the Human Metabolome Database HMDB^35^ and the LIPID MAPS Structure Database (LMSD)^36^ within a mass tolerance of 10 ppm. The removal of metabolites with intensities influenced by the measurement order, experimental batch effects, and postmortem delay yielded 1366 confounder-free metabolic peaks detected in both ionization modes (Fig. 1a, Supplementary Data 2 and 3). Multidimensional scaling (MDS) of the ASD and control samples using normalized intensities of these 1366 metabolites revealed a separation of very young individuals from the rest (Fig. 1b). Correspondingly, age explained 25% of the total metabolic variation among samples, while ASD accounted for 10%, postmortem delays (PMD) accounted for 8%, and other factors such as sex, sample quality, and ethnicity accounted for <5% each.

### Metabolic changes in ASD

**Fig. 2:**
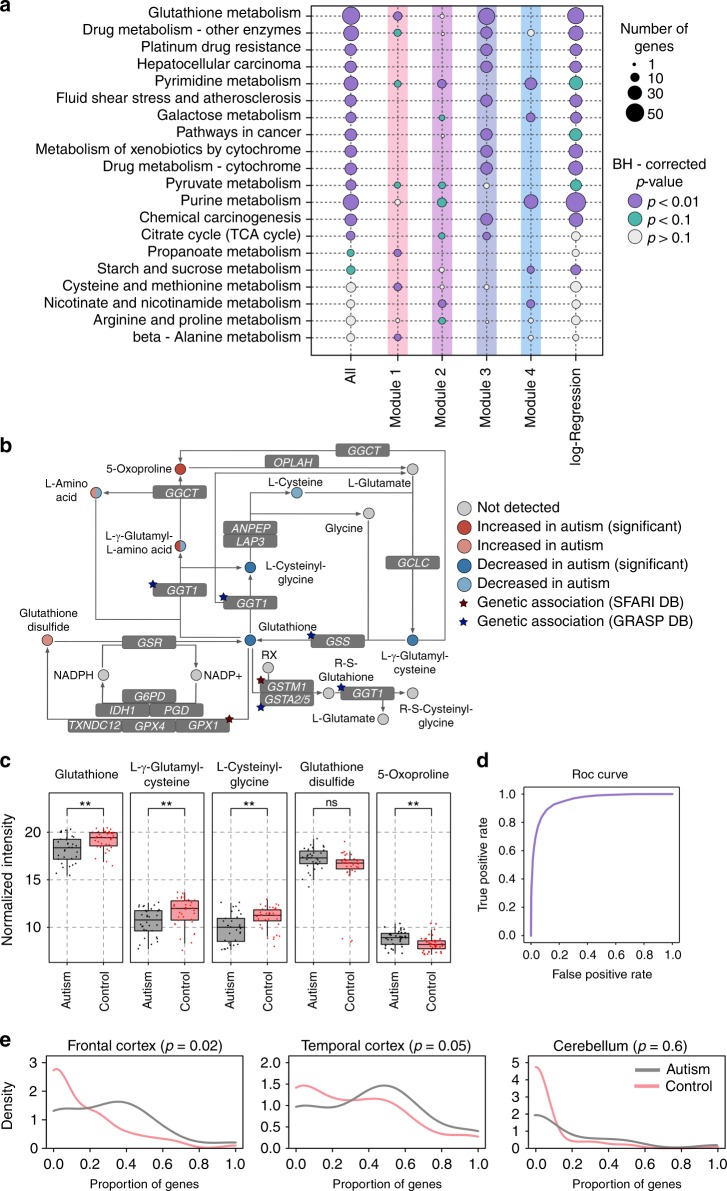
Characterization of metabolite intensity differences identified in the prefrontal cortex of ASD individuals. **a** Summary of top functional pathways enriched in genes linked to metabolites represented in different categories using KEGG annotation. The categories include: all 202 ASD-related metabolites identified using ANCOVA (All); ASD-related metabolites within each module (modules 1–4); and ASD metabolic predictors identified using logistic regression (log-regression). The size of each circle is proportional to the number of genes within the pathway linked to metabolites in a given category (smaller circles correspond to a smaller number of genes). The color of each circle indicates the BH-corrected enrichment *p*-values. **b** Simplified schematic representation of the glutathione pathway based on the KEGG annotation. Circles represent metabolites. Circle colors indicate the direction and significance of the difference. The double coloring of L-γ-glutamyl-L-amino acid and L-amino acid represent intensity changes of different compounds that fall under this putative annotation. Stars mark genes containing genetic variants associated with the ASD according to SFARI and GRASP databases^49,50^. **c** The intensities of five metabolites from the glutathione metabolism pathway showing differences in ASD: glutathione, L-γ-glutamylcysteine, L-cysteinylglycine, glutathione disulfide, and 5-oxoproline. Boxes show the first and the third quartiles and the median of the data; the whiskers extend to the minimum and maximum data values located within 1.5 interquartile range from the box. Dots indicate actual intensity values for individual samples. Colors represent ASD individuals (gray) and control individuals (red). Stars indicate the significance of differences between metabolite intensity in ASD individuals and controls (ANCOVA, BH-corrected *p*-value: ** < 0.01; ns > 0.01). **d** Average area under the receiver operating characteristic curve (ROC AUC) calculated using logistic regression with optimal parameters performed 500 times on different subsets of the individuals. **e** Distributions of the proportions of genes showing expression difference in ASD (|log2 fold change|>0.2) among genes linked to each of 202 ASD-related metabolites (gray curve) and the other metabolites detected in our study (red curve). Expression data was taken from ref. ^40^. Panels show the distribution in three brain regions: left—frontal cortex, center—temporal cortex, right—cerebellum

Of the 1366 detected metabolites, 202 (15%) showed significant intensity differences between ASD and control samples (ASD-related metabolites, ANCOVA, BH-corrected *p* < 0.05, nominal *p* < 0.009). The unsupervised clustering of the temporal intensity profiles of these 202 metabolites revealed four modules (Fig. 1c). Genes linked to the ASD-related metabolites using KEGG annotation were significantly overrepresented in a total of 16 pathways (the Kyoto Encyclopedia of Genes and Genomes^37^) (hypergeometric test, BH-corrected *p* < 0.05, nominal *p* < 0.008, Fig. 2a, Supplementary Data 4 and 5). Of the 16 enriched pathways, 10, including glutathione metabolism, were identified at a similar significance level using 42 metabolites from the module 3, characterized by an intensity decrease in ASD, especially during the first 20 years of life. Notably, the glutathione metabolism pathway contained not only significant intensity differences of glutathione and linked metabolites, but also genetic variants reported to be associated with ASD in enzymes catalyzing the corresponding reactions, such as *GPX1*, *GSTM1*, *GGT1*, and *GSS* (Fig. 2b, c). The concentrations of glutathione and related metabolites display technical variability, for instance after long-term storage of metabolite extracts (Norris et al. 2001)^38^. In our study, however, the intensities of the corresponding metabolites remained stable in brain samples with lengthy postmortem delay (Supplementary Fig. 1). Module 4, which contained 52 metabolites, was enriched in four of the 16 pathways, including strong enrichment in purine and pyrimidine metabolism pathways. By contrast, modules 1 and 2, characterized by elevated intensities in ASD, were not substantially overrepresented within the 16 pathways, but were enriched in the four additional module-specific pathways, including amino acid and nicotinamide metabolism.

### Metabolic predictors of ASD

Metabolite intensity differences between ASD cases and unaffected controls might allow for the classification of PFC samples of ASD individuals as a distinct group using machine-learning algorithms. To test this, we constructed a predictive model based on logistic regression with lasso regularization to assign each sample to the ASD or control group using the metabolite intensities. We then performed stability selection^39^, a procedure based on subsampling, to rank the metabolites’ contribution to the model and to assess the accuracy (Supplementary Fig. 2). Remarkably, the model distinguished ASD and control cases with more than 95% accuracy, estimated as the area under the ROC curve (ROC AUC), which corresponds to the area under the curve mapping the true positive rate (sensitivity) to the false positive rate (1-specificity) for different discrimination thresholds (Fig. 2d). Thus, the AUC ROC provides a measure of the diagnostic ability of the classifier.

The model’s metabolic predictors overlapped significantly with metabolites showing significant intensity differences in ASD in the ANCOVA analysis (Fisher test, *p* < 0.001). Consistently, KEGG pathways enriched in the top 200 metabolic predictors were in good agreement with pathways obtained in the ANCOVA analysis, with glutathione metabolism and purine metabolism occupying the top positions for each of the two methods (Fig. 2a, Supplementary Data 4).

### Gene expression associated with metabolic changes in ASD

To test whether the metabolite intensity differences between ASD and control cases might be caused by gene expression differences, we examined data from three brain regions: frontal cortex, temporal cortex, and cerebellum, measured in 19 ASD individuals and 17 controls, including 15 ASD individuals and 5 controls from the present study^40^. In two cortical regions, but not in the cerebellum, genes linked to ASD-related metabolites indeed showed significantly more expression differences between ASD individuals and controls than genes linked to metabolites showing no intensity difference in ASD (KS-test, *p* < 0.05, Fig. 2e, Supplementary Data 5 and 6).

### Evolution of metabolic changes in ASD

**Fig. 3:**
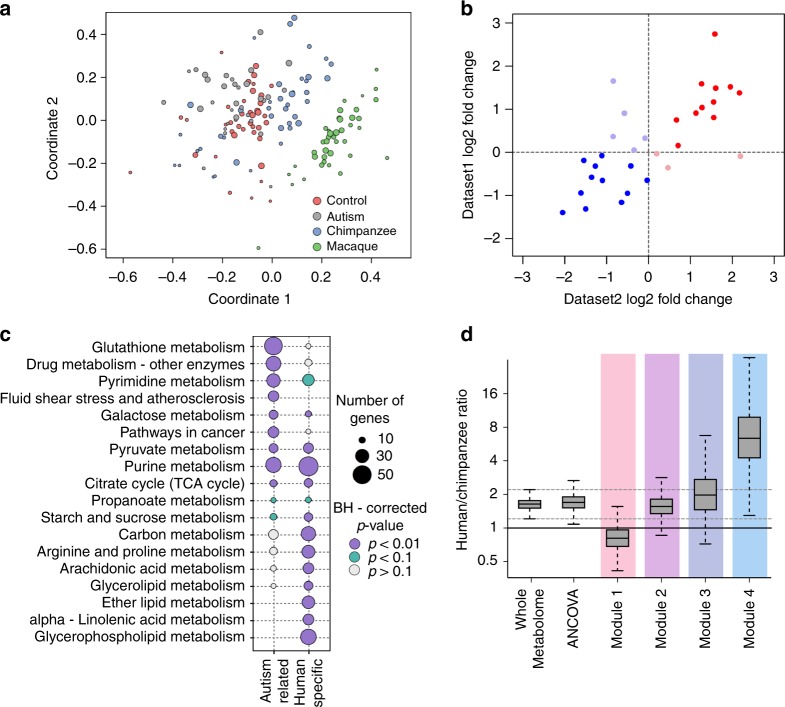
Evolution of metabolite intensity differences identified in the prefrontal cortex of ASD individuals. **a** The relationship among species and individuals plotted as the first two dimensions of the multidimensional scaling (MDS) procedure based on intensities of 1366 metabolites. Circles represent individual samples. Colors represent: ASD individuals (gray), control human individuals (red), chimpanzees (blue), and macaques (green). The size of each circle is proportional to the individual’s age (smaller circles correspond to younger ages). **b** Metabolite intensity differences between humans and macaques measured using our data (Dataset 1) and a published dataset (Dataset 2)^41^. The intensity differences were calculated as log2-transformed differences between the average intensity values within each species. Dots represent individual metabolites detected in both datasets (*n* = 31). Colors indicate plot quadrants. **c** Summary of top functional pathways enriched in genes linked to metabolites represented in two categories using KEGG annotation. The categories include: all 202 ASD-related metabolites identified using ANCOVA (ASD-related) and human-specific metabolites (human-specific). The size of each circle is proportional to the number of genes within the pathway linked to metabolites in a given category (smaller circles correspond to a smaller number of genes). The color of each circle indicates BH-corrected enrichment *p*-values. **d** The ratio of human-specific and chimpanzee-specific metabolites represented in different categories: all 1366 detected metabolites (Whole Metabolome); all 202 ASD-related metabolites identified using ANCOVA (ANCOVA); and ASD-related metabolites within each module (modules 1–4). Boxes show the first and the third quartiles and the median of the data, the whiskers extend to the minimum and maximum data values located within 1.5 interquartile range from the box. Dashed gray lines indicate the inter-whisker range of the detected metabolites

Previous work linked gene expression differences in ASD to developmental gene expression features unique to humans^4^. To assess this link at the metabolite level, we analyzed metabolite intensities in the PFC of 40 chimpanzees with ages between 0 and 42 years, and 40 rhesus macaques with ages between 14 weeks post-conception and 21 years (Supplementary Data 1). The chimpanzee and macaque samples were measured together with human control and ASD samples in a random order. A computational analysis of nonhuman primates, conducted in parallel with the human samples, yielded intensities in chimpanzee and macaque samples for 1366 metabolites confidently detected in human dataset. A multidimensional scaling (MDS) analysis based on the intensities of these metabolites revealed predominant sample separation according to age and species identity (Fig. 3a).

The identification of significant metabolite intensity differences and their placement on the evolutionary lineages (ANCOVA, BH-corrected *p* < 0.05, nominal *p* < 0.01) revealed 170 human-specific and 55 chimpanzee-specific differences defined using strict criteria, and 756 human-specific and 410 chimpanzee-specific differences defined using relaxed criteria. Human-specific intensity differences detected in this study agreed well with the differences calculated using the published metabolome dataset (Fig. 3b, Pearson correlation *R* = 0.71, *p* < 0.01)^41^.

Genes linked to human-specific metabolites were significantly overrepresented in a total of 27 KEGG pathways (hypergeometric test, BH-corrected *p* < 0.05, nominal *p* < 0.015, Supplementary Data 4). Notably, these pathways overlapped significantly with pathways enriched in ASD-related metabolite intensity differences, and included purine, pyrimidine, and pyruvate metabolism (Fisher test, *p* < 0.01, Fig. 3c).

While the ratio of human-specific metabolite intensity differences to the chimpanzee-specific ones among 202 ASD-related metabolites coincided with the one for all detected metabolites, their distribution among the four ASD modules was not uniform. Module 1 contained fewer human-specific metabolite intensity differences compared with the average, while module 4 contained approximately five times more (Wilcoxon test, *p* < 0.01, Fig. 3d). Notably, module 4 was particularly enriched in metabolic pathways overlapping between ASD-related and human-specific differences, including purine and pyrimidine metabolism (Fisher test, *p* < 0.1). To assess whether this excess of human-specific differences is due to a smaller number of compounds contained in module 4, we repeated analysis randomly sampling 30 metabolites from each module (Supplementary Fig. 3). While this procedure increased variation in all modules, module 4 retained an excess of human-specific metabolite intensity differences compared with the other modules (Wilcoxon test, *p* < 0.01).

## Discussion

In this study, we show that the metabolite composition of the gray matter of the prefrontal cortex differs substantially between ASD individuals and healthy controls. Despite the moderate statistical power of the study, as many as 15% of metabolites present in the prefrontal cortex alter their intensities significantly in ASD. These metabolites form specific age-dependent intensity patterns and cluster in specific KEGG pathways.

It is noteworthy that of the 16 pathways altered in the prefrontal cortex of ASD individuals based on ANCOVA results, 10 were reported in studies analyzing metabolite abundance of urine and blood samples, which is significantly more than expected by chance (Fisher test, *p*-value < 0.05). These pathways include glutathione metabolism, purine metabolism, pyruvate metabolism, propanoate metabolism, TCA cycle, galactose metabolism, starch and sucrose metabolism, nicotinate and nicotinamide metabolism, cysteine and methionine metabolism, and arginine and proline metabolism. Metabolite intensity differences clustering in these 10 pathways were all reported in the urine of ASD individuals^11–22^. Metabolite intensity differences clustering in the TCA cycle, glutathione metabolism, and pyruvate metabolism pathways were further reported in the blood of ASD individuals^23,24,27,31,33^. Intensity differences of individual metabolites, such as glutathione and cysteine decrease, as well as a glutathione disulfide and adenosine intensity increase in ASD individuals identified in our study (Fig. 2c, Supplementary Fig. 4), were similarly reported in blood^33^. Furthermore, the same direction and magnitude of the relative intensity differences between ASD individuals and controls were observed for glutathione and glutathione disulfide in the cerebellum and temporal cortex^42^. Similarly, intensity differences of 3-methoxytyramine and 5,6-dihydrouridine that increased in the cerebellum of ASD individuals^25^ are detected and reproduced in our dataset (Supplementary Fig. 5). At the gene expression level, the differences reported in the neocortex of ASD individuals^40^ are greater for genes linked to ASD-related metabolites found in our study compared with genes linked to the other detected metabolites. Taken together, these observations support the previous results and indicate a general agreement between ASD-related metabolite intensity differences detected in the brain and differences detected in the blood and urine analyses. Purine metabolism is particularly interesting, as purinergic signaling is involved in neurodevelopment processes, including cell proliferation, differentiation and neuron-glia cross-talk^43,44^. Moreover, purinergic signaling was shown to be altered in multiple psychiatric disorders, including ASD^45^.

Currently, to the best of our knowledge, no studies have investigated autism-associated metabolic alterations in brain and blood, neither in patients nor in animal models. More generally, few studies addressing the relationship between metabolic alterations in brain and blood yield different conclusions. The Alzheimer disease study conducted in nearly 1000 individuals revealed parallel alterations in brain and blood for several of the 26 metabolites linked with the diagnosis^46^. Similarly, stress-induced changes in the neurotransmitter concentration levels in the mouse brain were also detected in serum^47^. By contrast, the metabolite analysis in the mouse model of Parkinson disease identified significant alterations in brain in 14 metabolic pathways, only one of which was also altered in blood^48^. Our data show the relationship between metabolic alterations in brain and blood at the pathway level, rather than at the level of individual metabolites. It is appealing to suggest that such a relationship might be caused by several key pathway intermediates capable of crossing the blood–brain barrier. Alternatively, the link between metabolic alterations in blood and brain could be caused by mutations in metabolic enzymes present in both tissues. Notably, GWAS studies indeed reported multiple mutations linked to ASD within genes encoding metabolic enzymes^49,50^.

Our analysis further yields a number of novel observations. First, we uncover novel metabolite intensity differences complementary to previously reported ones. For instance, within the glutathione pathway, we show that in addition to a glutathione intensity decrease, two other metabolites, L-cysteinylglycine and L-γ-glutamyl-L-cysteine, display intensity differences in ASD. Notably, enzymes catalyzing reactions involving glutathione, L-cysteinylglycine, and L-γ-glutamyl-L-cysteine were shown to contain genetic variants previously linked to ASD, including polymorphisms in such genes as *GPX1*, *GSTM1*, *GGT1*, and *GSS*^51–53^.

Second, we identify a number of metabolic pathways that were not previously linked to ASD, including pyrimidine metabolism, beta-alanine metabolism, and three pathways related to drug metabolism by cytochromes. Metabolic processes in these pathways are connected to the reported ones. For instance, changes in pyrimidine metabolism might be linked to purine metabolism alterations, as both pathways involve the essential precursors for RNA and DNA synthesis and are well interconnected. Furthermore, pyrimidine nucleotides are involved in polysaccharide and phospholipid synthesis, detoxification processes, as well as proteins’ and lipids’ glycosylation^54^. Notably, functional deficiency of pyrimidine pathway genes, such as uridine monophosphate synthase (*UMPS*), dihydropyrimidine dehydrogenase (*DPYD*), Dihydropyrimidinase (*DPYS*), beta-ureidopropionase 1 (*UPB1*), leads to neurological aberrations, including ASD-like features in case of DPD deficiency (*DPYD* gene deficiency)^55^. Metabolic changes in the beta-alanine pathway might be linked to the pyrimidine metabolism pathway, as the main sources of beta-alanine include the catabolism of cytosine and uracil. Moreover, beta-alanine intensity is decreased in DPD (*DPYD* gene deficiency), DHP (*DPYS* gene deficiency), and BUP-1 (*UPB1* gene deficiency) induced pyrimidine pathway deficiencies^55^. In addition, key enzymes participating in cytochrome mediated drug metabolism are also involved in the glutathione metabolism pathway.

Third, we show that machine-learning algorithms can accurately identify metabolic features characteristic of ASD individuals represented in our dataset. The metabolites selected by the predictive model cluster in 14 of the 16 pathways discovered using ASD-related metabolites identified by ANCOVA and overlap with eight pathways reported in urine and blood samples. It is important to note that our study does not involve individuals with other cognitive disorders, which share comorbidities with some of ASD individuals, such as schizophrenia^56^. Therefore, our predictive model might include metabolite abundance alterations shared among disorders.

Fourth, by conducting brain metabolome measurements in nonhuman primates, we show that ASD-related metabolites falling within module 4 contain an almost eightfold excess of metabolite intensity differences unique to humans, compared with the chimpanzee-specific differences. Accordingly, a number of pathways enriched in ASD-related metabolite intensity differences, including purine, pyruvate metabolism, TCA cycle, and galactose metabolism, also showed an excess of human-specific metabolite intensity differences. This result is notable, given that ASD tends to affect cognitive abilities particularly pronounced in humans^4^. Generally, we find almost three times more metabolite intensity differences specific to humans, compared with chimpanzees. These results agree with previously reported excess of human-specific metabolite concentration differences in PFC^41^ and align well with the hypothesis postulating disruption of recently evolved cognitive mechanisms underlying communication and socialization in ASD^1–4^.

The main limitations of our study include its relatively small sample size, caused by the limited availability of brain tissue samples from ASD individuals. Accordingly, we can only estimate general traits present in the majority of individuals and cannot assess the presence of discrete molecular endophenotypes of ASD. Yet, the differences we detect correlate with disease severity, specifically with Autism Diagnostic Interview-Revised (ADI-R) score (Supplementary Fig. 6), indicating the possible connection between the metabolic alterations and the disorder manifestations. Importantly, the intensity patterns of modules were not driven by cases with extreme ADI-R scores: modules based only on individuals with moderate ADI-R scores closely reproduced all reported abundance profiles (Supplementary Fig. 7). Our results clearly highlight the need for further studies of metabolite intensity differences in autism involving more ASD individuals, as well as individuals affected by other common cognitive disorders to assess the disease-specificity of the alterations.

Our study demonstrates that the metabolic changes detected in blood might be informative of the metabolite intensity changes taking place in the brain. This result opens an opportunity for the design of ASD diagnostic tools based on the concentration measurements for a limited set of informative metabolites in blood or urine samples. The creation of reliable and objective ASD diagnostic tests would greatly facilitate the medical treatment of patients and selection of better-customized treatment routines. Furthermore, it would open the door for widespread metabolic biomarker-based clinical practices, providing the collection of standardized metabolic data from a large number of individuals. This, in turn, would create an essential resource for the construction of complex predictive models, leading to improved specificity in disease classification and treatment selection. Finally, better knowledge of the molecular changes taking place in the disease will lead to the more rapid development of better medical treatments. It should be mentioned, however, that design of such diagnostic assays would require extensive validation using independent sets of samples, and preferably include data for the brain, blood, and urine samples collected using the same experimental, analytical, and statistical frameworks.

## Methods

### Samples

This study was reviewed and approved by the Institutional Animal Care and Use Ethics Committee at the Shanghai Institute for Biological Sciences, CAS. Informed consent for the use of human tissues for research was obtained in writing from all donors or their next of kin. All nonhuman primates used in this study suffered sudden deaths for reasons other than their participation in this study and without any relation to the tissue used.

We used PFC samples dissected from the frozen postmortem brains of 40 cognitively unaffected human controls (0–62 years old), 32 ASD cases (2–60 years old), 40 chimpanzees (0–43 years old), and 40 rhesus macaques (0–21 years old). Special care was taken to dissect gray matter only.

Control human samples were obtained from the NICHD Brain and Tissue Bank for Developmental Disorders at the University of Maryland, USA, the Maryland Brain Collection Center, Maryland, USA, and the Harvard Brain Tissue Resource Center. ASD samples were obtained from the NICHD Brain and Tissue Bank for Developmental Disorders and the Harvard Brain Tissue Resource Center. All the control and ASD brain samples used in this study were also part of recently published transcriptomic^4^ and lipidomic^56^ studies.

Chimpanzee samples were obtained from the National Chimpanzee Brain Resource (NS092988), the Alamogordo Primate Facility, New Mexico, USA, the Anthropological Institute and Museum of the University of Zürich-Irchel, Switzerland, the Biomedical Primate Research Centre, the Netherlands, Department of Anthropology, The George Washington University, Washington, DC, and Burgers’ Zoo in Arnhem, the Netherlands. Rhesus monkey samples were obtained from the Suzhou Experimental Animal Center, China. PFC dissections were made from the frontal part of the superior frontal gyrus. For all samples we preferentially dissected and analyzed gray matter material.

To identify metabolites affected by postmortem delay in primates, we additionally collected two rhesus macaque samples, dissected 5–6 h after death. Other macaque samples used in this study had postmortem delay (PMD) lower than 20 min.

### Sample preparation

Metabolites were extracted from frozen tissue powder using a methanol: methyl-tert-butyl-ether (1:3 (vol/vol)) solution as described in ref. ^57^. In brief, 10–15-mg tissue samples were dissected on dry ice from the frozen tissue without thawing. Dissected samples were weighed and transferred to pre-cooled 2 ml round bottom reinforced Precellys tubes containing 2.8 mm. zirconia beads. After the addition of a fixed volume (0.5 ml) of pre-cooled (−20 °C) extraction buffer to each tube, we performed two cycles of homogenization using the following parameters: stirring intensity 5000 rpm, homogenization temperature 7 °C, cycle time 45 s, and 15 s break. Several blank extraction samples were added to the end of each batch (48 samples). The blank samples were represented by the same 2 ml round bottom reinforced Precellys tubes without any sample material subjected to all extraction procedures. After the addition of a fixed volume (0.5 ml) of extraction buffer, the homogenates were vortexed and incubated for 30 min at 4 °C on an orbital shaker followed by a 10-min ultra-sonication in an ice-cooled sonication bath. For each sample, the homogenate was transferred to a pre-labeled 2 ml Eppendorf tube followed by the addition of 700 μl of an H_2_O:methanol (3:1 (vol/vol)) solution containing 0.7 μg of corticosterone and ampicillin. Finally, to separate the organic phases from aqueous phases and to precipitate proteins, after a brief vortexing, the homogenates were centrifuged for 10 min at 14,000 × *g* at 4 °C. Subsequently, 300 μl of the lower aqueous phase containing hydrophilic compounds were transferred to a pre-labeled 1.5 ml Eppendorf tube and the solvent was evaporated using a speed vacuum centrifuge at room temperature. Dry metabolic extracts were stored at −80 °C prior to MS analysis. For quality control, we further constructed a pooled sample composed by the aliquots of metabolite extracts from all samples used in the analysis.

### Mass spectrometry analysis

The dried extracts were resuspended in 200 µl of ice-cold 20% aqueous solution of acetonitrile prior to MS analysis. After a brief rigorous vortexing, the samples were incubated for 30 min at 4 °C on an orbital shaker followed by a 10 min ultra-sonication in an ice-cooled sonication bath and centrifugation for 10 min at 14,000 × *g* at 4 °C. For the MS analysis, 40 µl of supernatant was transferred to 350 μl autosampler glass vials (Glastechnik Grafenroda, Germany). A chromatography separation of metabolites prior to MS was performed using Acquity I-Class UPLC system (Waters, UK). Metabolites were separated on a normal phase unbounded silica column RX-SIL (100 mm × 2.1 mm, 1.8 µm, Agilent, USA) coupled to a guard precolumn with the same phase parameters. We used two mobile phases for the chromatographic separation. Buffer A was water containing 10 mM ammonium acetate and 0.2 mM ammonium hydroxide in water:acetonitrile (95:5 (vol/vol)) solution (pH value 8.0), and buffer B was 100% acetonitrile. The gradient separation was 0 min 0% A, 0.01–10 min linear gradient from 0 to 100% A, 10–14 min 100% A, 14–17 min linear gradient from 100 to 0% A, and 17–25 min 0% A. After a 3-min wash with 100% buffer A, the column was re-equilibrated with 100% buffer B. The flow rate was set to 500 µl/min. The column temperature was maintained at 40 °C. The mass spectra were acquired in positive and negative mode using a heated electrospray ionization source in combination with Q Exactive Hybrid Quadrupole-Orbitrap mass spectrometer (Thermo Scientific, Germany). Negative ion mode samples were run after the positive ion mode cohort with 6 µl injection of non-diluted samples. Spray voltage was set to 4.5 kV in positive mode and to 3 kV in negative mode. The remaining MS settings were the same in both ionization modes: S-lens RF level—70; heated capillary—250 °C; aux gas heater temperature—350 °C; sheath gas flow rate—45 arbitrary units; aux gas flow rate—10 arbitrary units; sweep gas flow rate—4 arbitrary units. Full scan resolutions were set to 70,000 at 200 *m*/*z*. The full scan target was 10^6^ with a maximum fill time of 50 ms. The spectra were recorded using full scan mode, covering a mass range from 100 to 1500 *m*/*z*. For quality control, pooled samples were injected four times prior to each ionization mode run to condition the column and after the completion of both ionization modes. In addition, a pooled sample was injected after every 48 sample injections to assess the sensitivity and retention time consistency of the system, sample reproducibility, and the compound stability over the time of the MS analysis.

### Mass spectrometry data processing

MS peaks obtained in positive and negative modes were aligned across samples as described in refs. ^57,58^. This procedure was applied to all samples, including control and ASD individuals, chimpanzees, and macaques. We used RefinerMS software (Version 6.0, GeneData, Basel, Switzerland) to generate a list of chromatographic peaks with associated *m*/*z*, retention time and intensity values. Putative annotation of metabolites was based on the computational matching of *m*/*z* values to the Human Metabolome Database (HMDB)^35^ and the LIPID MAPS Structure Database (LMSD)^36^ with a mass tolerance of 10 ppm, allowing [M + H], [M + NH4], and [M + Na] modifications as possible adducts in positive ionization mode, and [M−H], [M + Formic acid-H], [M-H_2_O-H], and [M + Na-2H] modifications in negative ionization mode^57,59^.

To eliminate the effects of the measurement order on metabolite intensities, we computed this effect for each metabolite by fitting a support vector regression model with a Gaussian kernel and one set of parameters for all metabolites to the log2 transformed, centered to the mean = 0, and scaled to standard deviation = 1 intensity values. The resulting functions were clustered using a k-means algorithm. After a visual evaluation, the clusters obviously affected by the measurement order were discarded. The intensity values of the remaining metabolites showing measurement order effect were corrected by subtracting the average of the corresponding cluster. The metabolite intensities were then recalculated to the original magnitude scale. After this procedure, a total of 1405 peaks remained. To correct for the variation related to the tissue weight, the metabolite intensities were normalized across samples using upper-quartile normalization separately in each mode.

To eliminate metabolites affected by a postmortem delay, we compared metabolite intensities in macaque PFC samples collected from 42 individuals of different ages with a short PMD of <20 min after death (40 individuals) and with a prolonged PMD of 5–6 h (two individuals). For each metabolite, we assessed the difference between intensities in samples obtained after prolonged PMD and the ones interpolated at the corresponding age from a spline curve fitted to the intensities measured in samples with a short PMD with four degrees of freedom. Metabolites with intensities in samples with prolonged PMD falling outside the 95% confidence interval of spline curve predictions were excluded from further analysis. This procedure excluded 39 (2.8%) of the 1405 metabolites (Supplementary Fig. 8).

### Statistics and reproducibility

The resulting 1366 metabolites were used to explore relationships among samples with a multidimensional scaling algorithm (MDS) (Figs. 1b, 3a). To estimate the variance explained by various factors, we applied principal variance component analysis (PVCA) as described in ref. ^4^.

To identify metabolites with intensity differences between ASD samples and unaffected controls, we used an analysis of covariance (ANCOVA) as described in refs. ^4,60^. Briefly, for each metabolite we chose the best polynomial regression model with age as a predictor and intensities as a response based on adjusted R^2^ criterion. Next, we used the F-test to evaluate whether the addition of disease/control status parameter significantly improved this model. The test was performed twice, using ASD samples as a reference for choosing the best polynomial regression model in one run, and control samples in the other. The resulting *p*-values were adjusted by the Benjamini-Hochberg (BH) approach. If the metabolite passed the BH-corrected *p*-value threshold of 0.05 in both cases, the compound was classified as an ASD-related metabolite.

To identify patterns of age-related intensity differences for ASD-related metabolites, we performed hierarchical clustering with 1 − Pearson correlation coefficient as the distance measure using both ASD and control samples. We used complete-linkage method of hierarchical clustering and cut the tree at four clusters (Fig. 1c). Segregation to a large number of clusters did not reveal novel patterns and yielded clusters with low numbers of metabolites (*n* < 20).

To test the overrepresentation of ASD-related metabolites in metabolic pathways, we performed a pathway enrichment analysis using R package clusterProfiler^61^ based on the enzymes that were directly linked to these metabolites according to the KEGG database annotation^37^. Genes directly linked to all the metabolites detected in our study were used as the background. The results were corrected for multiple testing using BH correction (Fig. 2a).

To test whether the disease status can be predicted using metabolite intensities, we implemented logistic regression with l_1_ regularization, which is a linear model with an additional penalty on its coefficients that makes it possible to train the model while simultaneously performing feature selection. Because the feature selection showed substantial variability among different training subsets of the data, we performed stability selection as described in refs. ^39^, which is a procedure based on multiple subsampling of the data. Briefly, the dataset was divided into the training and test sets and the model was trained using the training set with a fixed parameters (C = 100). For each trained model, we tested the performance of the classifier using a test set. By combining the results of all the data subsampling combinations (*n* = 500), we calculated the mean performance and, for each metabolite, the empirical probability of being incorporated into the model with a non-zero coefficient (Fig. 2d). We then ranked the metabolites according to this empirical probability. Based on model performance assessment, we chose a cutoff of the top 200 metabolites to define the set of model predictors.

We used two sets of cutoffs (stringent and relaxed) to identify species-specific metabolite intensity differences. To identify differences using the stringent cutoff, we used the ANCOVA approach described in refs. ^4,60^ for each species pair twice, using either species as a reference. If the test was significant for both human/chimpanzee and human/macaque pairs but not significant for the chimpanzee/macaque pair, then the metabolite was classified as showing human-specific intensity difference. Similarly, if the test was significant for both human/chimpanzee and chimpanzee/macaque pairs, but not significant for the human/macaque pair, then the metabolite was classified as showing chimpanzee-specific intensity difference. To conduct age alignment between species, we used the age scaling procedure described in ref. ^62^. To identify differences using the relaxed cutoff, we calculated distances between species using macaque metabolite intensities as a baseline. A metabolite was classified as showing human-specific intensity difference if its human-macaque distance was larger than chimpanzee-macaque distance, and the direction of changes relative to the macaque coincided in humans and chimpanzees. Similarly, a metabolite was classified as showing a chimpanzee-specific intensity difference if its chimpanzee-macaque distance was larger than human-macaque distance, and the direction of changes relative to macaque coincided between humans and chimpanzees. For each metabolite, the distance was calculated as the absolute difference between average z-transformed intensities within species. To assess human-specificity in each module, we performed 1000 subsamplings of the same number of samples (*n* = 30) from each of the three species and calculated the ratio of human-specific to chimpanzee-specific intensity differences for each subsampling (Fig. 3d).

We tested the overrepresentation of human-specific metabolite intensity differences defined using stringent criteria in KEGG pathways using R package clusterProfiler^61^ as described above. To test the significance of the overlap between ASD-related and human-specific pathways, we performed Fisher’s exact test (Fig. 3c).

To compare ASD-related metabolites with genes differentially expressed in ASD, we estimated gene expression levels using published dataset deposited in the Gene Expression Omnibus (GEO) under accession number GSE28521^40^. The dataset contains transcriptome data for 19 ASD samples and 17 control samples from three brain regions: the cerebellum, the frontal cortex, and the temporal cortex. To test whether genes linked to ASD-related metabolites tended to show an excess of expression levels particular to ASD, we calculated the proportion of genes with ASD-dependent expression linked to each metabolite. Genes showing absolute log2 fold change between the average expression of ASD samples and control samples >0.2 were defined as genes with ASD-dependent expression in this analysis (Fig. 2e).

To compare intensities of metabolites detected in our study with a previously published dataset of metabolite concentrations in humans, chimpanzees, and macaques^41^, we matched metabolites between the datasets using putative annotation. We then calculated log2 fold changes between the average metabolite intensities in humans and the average metabolite intensities in macaques (Fig. 3b). To test whether log2 fold change values agreed between datasets, we calculated the Pearson correlation coefficient and performed Fisher’s exact test.

To compare metabolome alterations in ASD detected in our study with published metabolite concentration changes in the urine and blood of ASD individuals, we performed Fisher’s exact test based on the overlap of KEGG metabolic pathways enriched in ASD-related metabolites in our study and in the union of published ones^11–24^.

### Reporting summary

Further information on research design is available in the Nature Research Reporting Summary linked to this article.

## Supplementary information

Supplementary Data 1

Supplementary Data 2

Supplementary Data 3

Supplementary Data 4

Supplementary Data 5

Supplementary Data 6

Supplementary Information

Description of additional supplementary items

Reporting Summary

## Data Availability

All data generated or analyzed during this study are included in this published article (and its supplementary information files).

## Code availability

Source code of software that has been written for data processing and analysis is freely available at https://cb.skoltech.ru/~khrameeva/autism/code/.
