# Supplementary material for: Metabolome signature of autism in the human prefrontal cortex
Source: Commun Biol. 2019 Jun 21;2:234. doi: 10.1038/s42003-019-0485-4 (PMC6588695; doi:10.1038/s42003-019-0485-4)
Supplement: Supplementary file 7 — Supplementary Information [file 42003_2019_485_MOESM7_ESM.pdf]

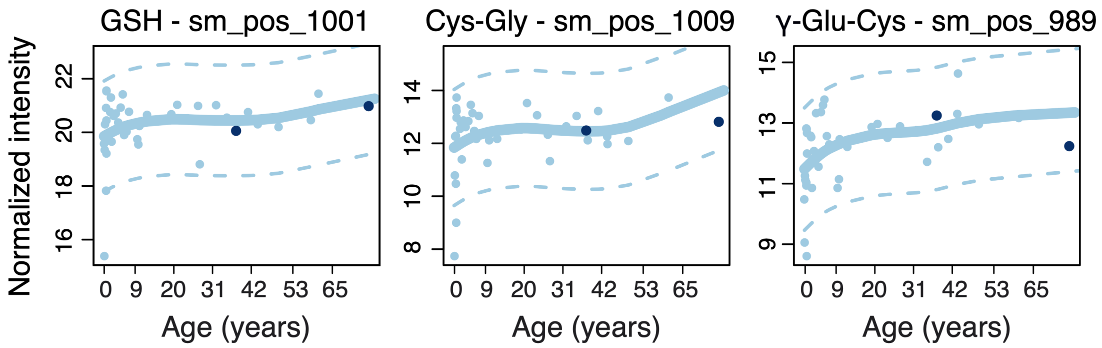

Supplementary Figure 1. Intensity profiles of compounds putatively annotated as glutathione (GSH) and two related metabolites, L-γ-Glutamyl-cysteine (γ-Glu-Cys) and L-Cysteinyl-glycine (Cys-Gly). The points indicate metabolite intensities in the PFC of macaque individuals with short post-mortem delays (<20 minutes, light blue) and long post-mortem delays (5-6 hours, dark blue). The lines represent the spline curves fitted with four degrees of freedom to the data points of short-postmortem-delay individuals. The dashed lines correspond to the 95% confidence interval of spline curve predictions.

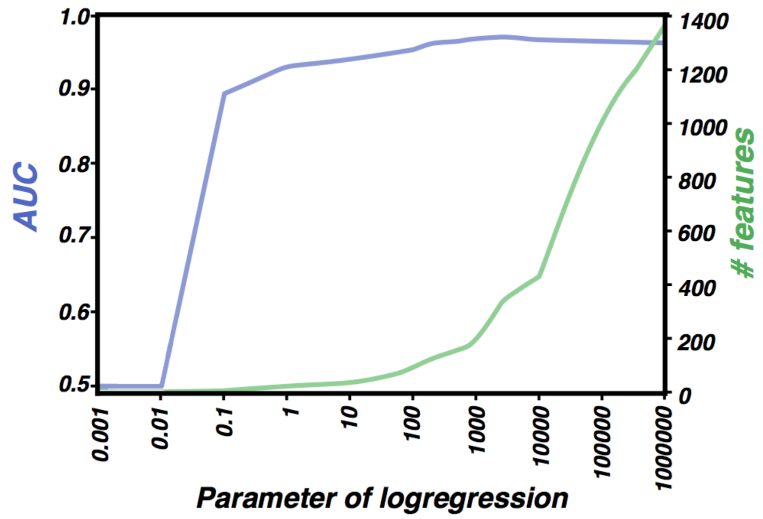

Supplementary Figure 2. Average area under the receiver operating characteristic curve (ROC AUC, left y-axis) and number of features selected by the model, depending on the regularization parameter of the model (# features, right y-axis). Both scales represent the average values of the parameters calculated over the multiple subsampling rounds performed during the stability selection procedure.

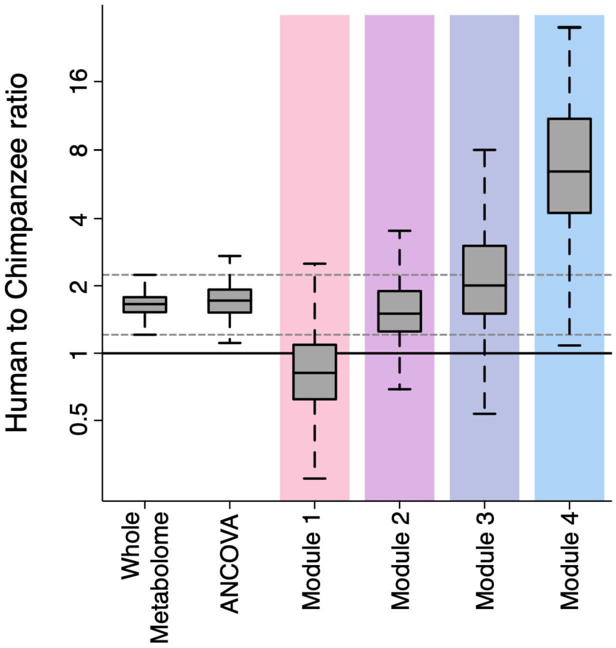

Supplementary Figure 3. The ratio of human-specific and chimpanzee-specific metabolites represented in different categories: all 1,366 detected metabolites (Whole Metabolome); 202 ASD-related metabolites identified using ANCOVA (ANCOVA); and 30 ASD-related metabolites randomly sampled from each of the four modules (Module 1-4).

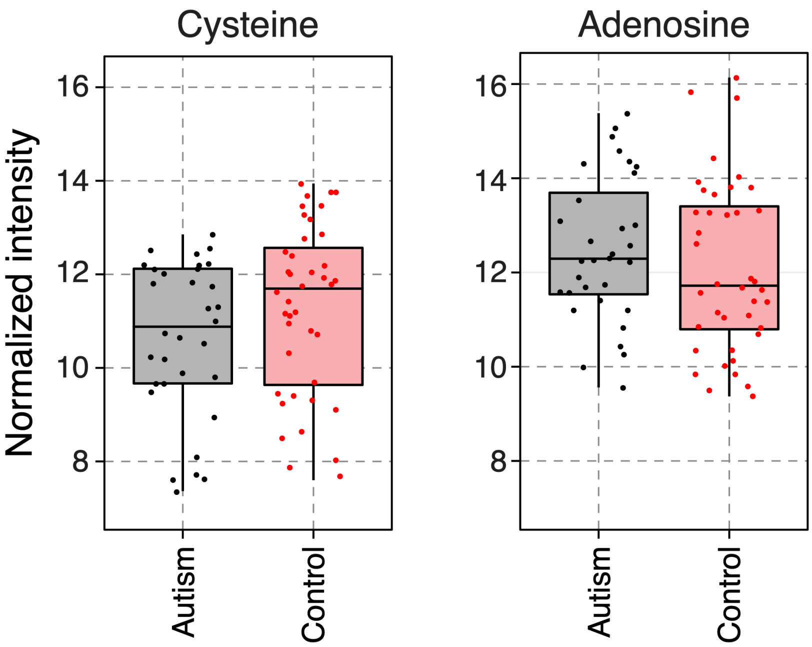

Supplementary Figure 4. Intensities of two metabolites in our data, cysteine and adenosine, reported as ASD-related in blood [1]. The boxes show the first and the third quartiles and the median of the data, while the whiskers extend to the minimum and maximum data values located within 1.5 interquartile range from the box. The dots indicate intensity values for individual samples. The colors represent ASD individuals (gray) and control individuals (red).

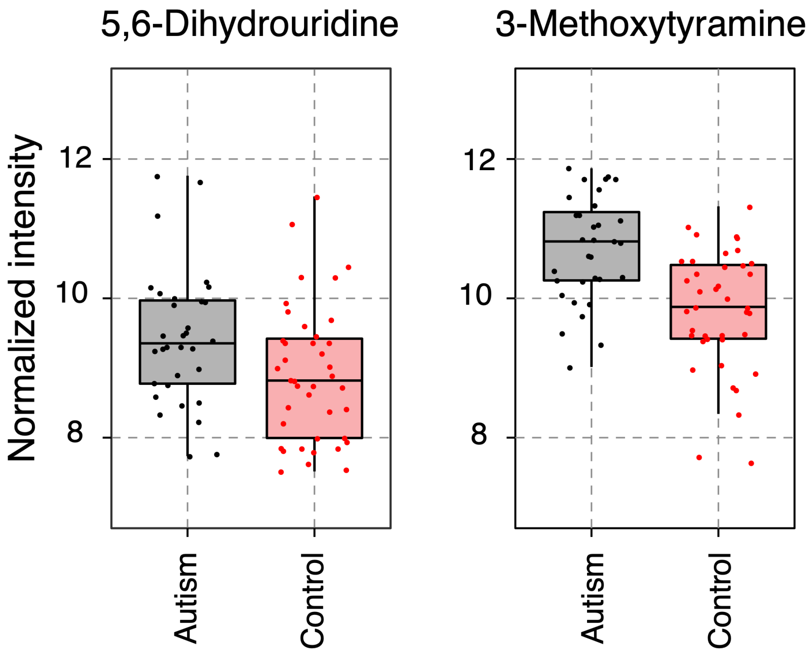

Supplementary Figure 5. Intensities of two metabolites in our data, 5,6-dihydrouridine and 3-methoxytyramine, reported as increased in the cerebellum of ASD individuals [2]. The boxes show the first and the third quartiles and the median of the data, while the whiskers extend to the minimum and maximum data values located within 1.5 interquartile range from the box. The dots indicate intensity values for individual samples. The colors represent ASD individuals (gray) and control individuals (red).

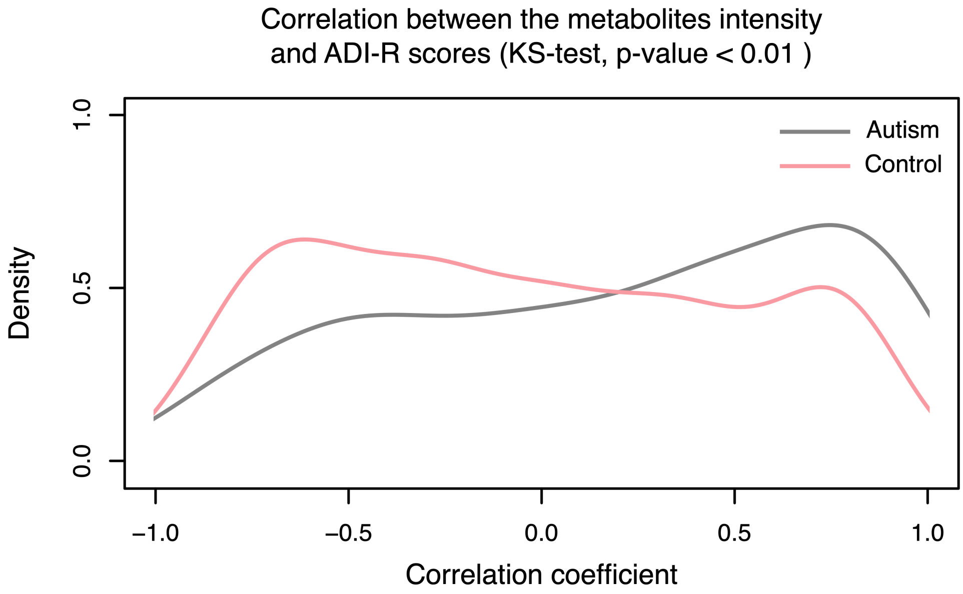

Supplementary Figure 6. Correlation between the average relative metabolite intensity and ADI-R scores for ASD-related metabolites (gray curve) and remaining metabolites (red curve).

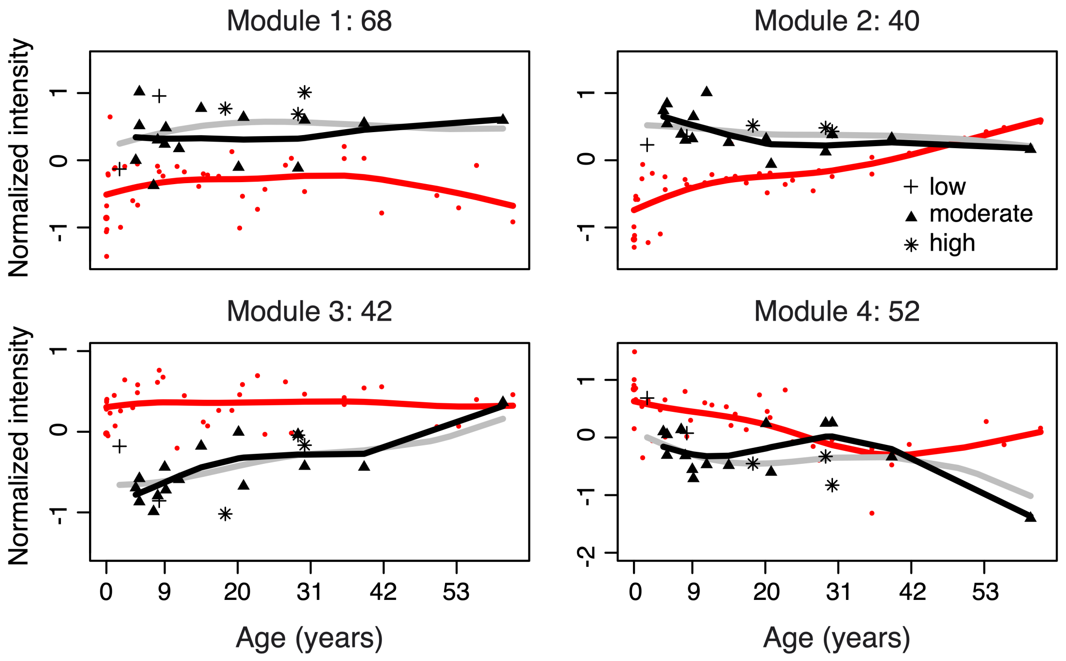

Supplementary Figure 7. Metabolite intensity patterns in the four modules showing different levels of the disease phenotype. The metabolite intensities within each module were standardized to mean = 0 and standard deviation = 1. Symbols represent individuals: red circles – controls; black stars – autism cases with high ADI-R scores; black triangles – autism cases with moderate ADI-R scores; black crosses – autism cases with low ADI-R scores. Lines represent cubic spline curves fitted to an individual's data (red – controls; gray – all ASD individuals; black – ASD individuals with moderate ADI-R scores).

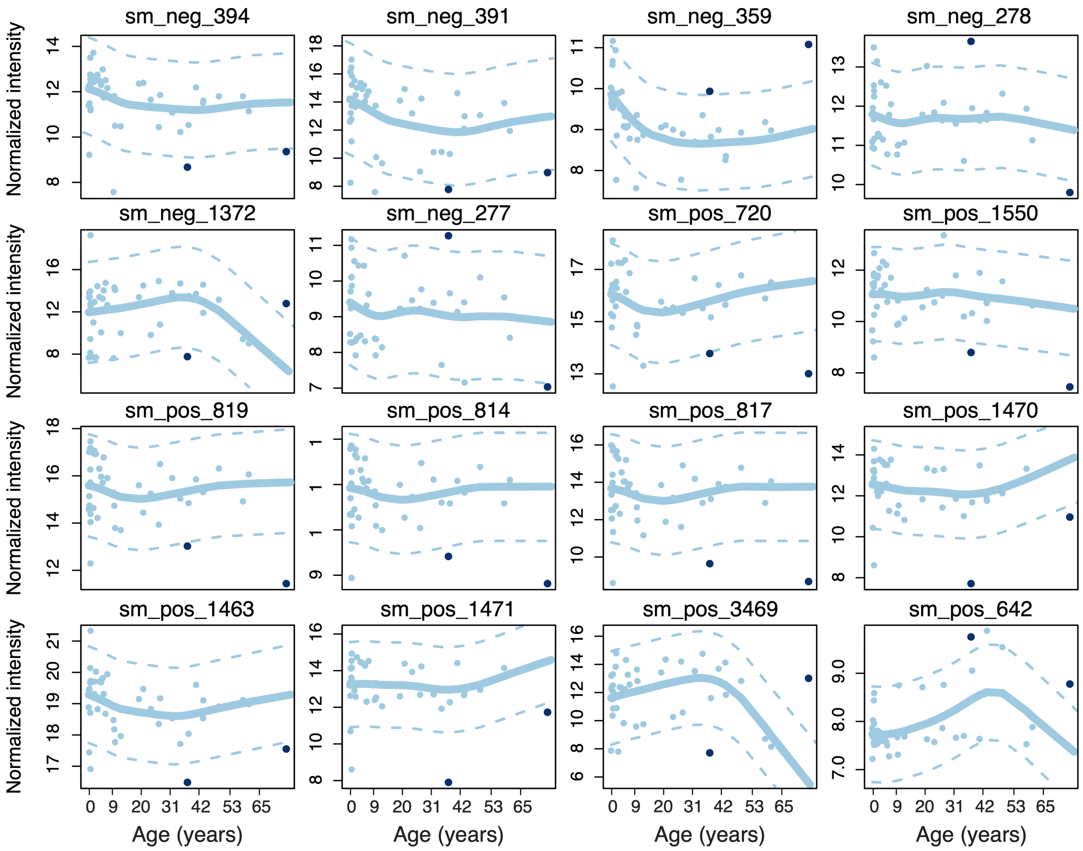

Supplementary Figure 8. Intensity profiles of 16 metabolites out of 39 metabolites classified as affected by postmortem delay in our analysis. The points indicate metabolite intensities in the PFC of macaque individuals with short postmortem delays (<20 minutes, light blue) and long postmortem delays (5-6 hours, dark blue). The lines represent the spline curves fitted with four degrees of freedom to the data points of short postmortem-delay individuals. The dashed lines correspond to the 95% confidence interval of spline curve predictions.

References

[1] James SJ, Cutler P, Melnyk S, Jernigan S, Janak L, Gaylor DW, et al. Metabolic biomarkers of increased oxidative stress and impaired methylation capacity in children with autism. The American Journal of Clinical Nutrition. 2004;80(6):1611-1617. doi:10.1093/ajcn/80.6.1611.

[2] Graham SF, Chevallier OP, Kumar P, Trkolu O, Bahado-Singh RO. High resolution metabolomic analysis of ASD human brain uncovers novel biomarkers of disease. Metabolomics. 2016;12(4). doi:10.1007/s11306-016-0986-9.
