# Supplementary material for: Metabolome signature of autism in the human prefrontal cortex
Source: Commun Biol. 2019 Jun 21;2:234. doi: 10.1038/s42003-019-0485-4 (PMC6588695; doi:10.1038/s42003-019-0485-4)
Supplement: Supplementary file 8 — Description of additional supplementary items [file 42003_2019_485_MOESM8_ESM.pdf]

Description of additional supplementary items

Supplementary Data 1. Sample information.

Supplementary Data 2. Intensity values of the 1,366 detected metabolites in the PFC of healthy humans, ASD individuals, chimpanzees, and rhesus macaques.

Supplementary Data 3. Putative annotation of detected metabolites.

Supplementary Data 4. Enrichment in KEGG pathways estimated using genes linked to ASD-related and human-specific metabolites.

Supplementary Data 5. Genes linked to the ASD-related metabolites based on KEGG annotation.

Supplementary Data 6. Differential expression analysis of ASD individuals and healthy controls samples from the transcriptome study [1] for genes linked to ASD-related metabolites.

References

[1] Voineagu I, Wang X, Johnston P, Lowe JK, Tian Y, Horvath S, et al. Transcriptomic analysis of autistic brain reveals convergent molecular pathology. Nature. 2011;474(7351):380-384. doi:10.1038/nature10110.
