# Supplementary material for: Metabolome signature of autism in the human prefrontal cortex
Source: Commun Biol. 2019 Jun 21;2:234. doi: 10.1038/s42003-019-0485-4 (PMC6588695; doi:10.1038/s42003-019-0485-4)
Supplement: Supplementary file 9 — Reporting Summary [file 42003_2019_485_MOESM9_ESM.pdf]

nature research

Corresponding author(s): Philipp Khaitovich

# Reporting Summary

Nature Research wishes to improve the reproducibility of the work that we publish. This form provides structure for consistency and transparency in reporting. For further information on Nature Research policies, see Authors & Referees and the Editorial Policy Checklist.

## Statistical parameters

When statistical analyses are reported, confirm that the following items are present in the relevant location (e.g. figure legend, table legend, main text, or Methods section).

| n/a | Confirmed | |
|---|---|---|
| ☐ | ☒ | The exact sample size (*n*) for each experimental group/condition, given as a discrete number and unit of measurement |
| ☐ | ☒ | An indication of whether measurements were taken from distinct samples or whether the same sample was measured repeatedly |
| ☐ | ☒ | The statistical test(s) used AND whether they are one- or two-sided<br>*Only common tests should be described solely by name; describe more complex techniques in the Methods section.* |
| ☐ | ☒ | A description of all covariates tested |
| ☐ | ☒ | A description of any assumptions or corrections, such as tests of normality and adjustment for multiple comparisons |
| ☐ | ☒ | A full description of the statistics including central tendency (e.g. means) or other basic estimates (e.g. regression coefficient) AND variation (e.g. standard deviation) or associated estimates of uncertainty (e.g. confidence intervals) |
| ☐ | ☒ | For null hypothesis testing, the test statistic (e.g. *F*, *t*, *r*) with confidence intervals, effect sizes, degrees of freedom and *P* value noted<br>*Give P values as exact values whenever suitable.* |
| ☒ | ☐ | For Bayesian analysis, information on the choice of priors and Markov chain Monte Carlo settings |
| ☒ | ☐ | For hierarchical and complex designs, identification of the appropriate level for tests and full reporting of outcomes |
| ☐ | ☒ | Estimates of effect sizes (e.g. Cohen's *d*, Pearson's *r*), indicating how they were calculated |
| ☐ | ☒ | Clearly defined error bars<br>*State explicitly what error bars represent (e.g. SD, SE, CI)* |

*Our web collection on statistics for biologists may be useful.*

## Software and code

Policy information about availability of computer code

| | |
|---|---|
| Data collection | We used RefinerMS software (Version 6.0, GeneData, Basel, Switzerland) to generate a list of chromatographic peaks with associated m/z, retention time and intensity values. |
| Data analysis | Source code of software that has been written for data processing and analysis is freely available at https://cb.skoltech.ru/~khrameeva/autism/code/ |

For manuscripts utilizing custom algorithms or software that are central to the research but not yet described in published literature, software must be made available to editors/reviewers upon request. We strongly encourage code deposition in a community repository (e.g. GitHub). See the Nature Research guidelines for submitting code & software for further information.

## Data

Policy information about availability of data

All manuscripts must include a data availability statement. This statement should provide the following information, where applicable:

- Accession codes, unique identifiers, or web links for publicly available datasets
- A list of figures that have associated raw data
- A description of any restrictions on data availability

All data generated or analysed during this study are included in this published article (and its supplementary information files).

# Field-specific reporting

Please select the best fit for your research. If you are not sure, read the appropriate sections before making your selection.

☒ Life sciences ☐ Behavioural & social sciences ☐ Ecological, evolutionary & environmental sciences

For a reference copy of the document with all sections, see nature.com/authors/policies/ReportingSummary-flat.pdf

# Life sciences study design

All studies must disclose on these points even when the disclosure is negative.

| | |
|---|---|
| Sample size | The sample size was not predetermined. Yet, the sample size was sufficient as we were able to identify 202 metabolites with statistically significant age-related intensity changes in ASD individuals compared to controls. |
| Data exclusions | No data were excluded from the analyses. |
| Replication | Experimental measurements were conducted in 40 control and 32 ASD individuals to insure reproducibility of our findings. |
| Randomization | The measurements were conducted in a random order using liquid chromatography coupled with mass spectrometry (LC–MS). |
| Blinding | The investigators were blinded to the group allocation during data collection, including metabolite extraction procedures and LC-MS measurements. |

# Reporting for specific materials, systems and methods

## Materials & experimental systems

| n/a | Involved in the study |
|---|---|
| ☒ | ☐ Unique biological materials |
| ☒ | ☐ Antibodies |
| ☒ | ☐ Eukaryotic cell lines |
| ☒ | ☐ Palaeontology |
| ☐ | ☒ Animals and other organisms |
| ☐ | ☒ Human research participants |

## Methods

| n/a | Involved in the study |
|---|---|
| ☒ | ☐ ChIP-seq |
| ☒ | ☐ Flow cytometry |
| ☒ | ☐ MRI-based neuroimaging |

## Animals and other organisms

Policy information about studies involving animals; ARRIVE guidelines recommended for reporting animal research

| | |
|---|---|
| Laboratory animals | Samples of 40 rhesus monkeys (0–21 years old) were obtained from the Suzhou Experimental Animal Center, China. All non-human primates used in this study suffered sudden deaths for reasons other than their participation in this study and without any relation to the tissue used. Details on sex and age of each individual are listed in Supplementary Data 1.<br>Samples of 40 chimpanzees (0–43 years old) were obtained from the National Chimpanzee Brain Resource (NS092988), the Alamogordo Primate Facility, New Mexico, USA, the Anthropological Institute and Museum of the University of Zürich-Irchel, Switzerland, the Biomedical Primate Research Centre, the Netherlands, Department of Anthropology, The George Washington University, Washington, DC, and Burgers' Zoo in Arnhem, the Netherlands. All non-human primates used in this study suffered sudden deaths for reasons other than their participation in this study and without any relation to the tissue used. Details on sex and age of each individual are listed in Supplementary Data 1. |
| Wild animals | The study did not involve wild animals. |
| Field-collected samples | The study did not involve samples collected from the field. |

## Human research participants

Policy information about studies involving human research participants

| | |
|---|---|
| Population characteristics | We used samples dissected from the frozen postmortem brains of 40 cognitively unaffected human controls (0–62 years old), 32 ASD cases (2–60 years old). Details on sex and age of each individual are listed in Supplementary Data 1. |

Recruitment

Control human samples were obtained from the NICHD Brain and Tissue Bank for Developmental Disorders at the University of Maryland, USA, the Maryland Brain Collection Center, Maryland, USA, and the Harvard Brain Tissue Resource Center. ASD samples were obtained from the NICHD Brain and Tissue Bank for Developmental Disorders and the Harvard Brain Tissue Resource Center. We obtained all samples that were available to us, without any selection except for the sample quality.
